# Does social capital aid in leveling the income gradient in child mental health? A structural analysis of the left-behind and not-left-behind Chinese children

**DOI:** 10.1186/s12889-023-16264-9

**Published:** 2023-07-20

**Authors:** Lijuan Gu, Linsheng Yang, Hairong Li

**Affiliations:** 1 Key Laboratory of Land Surface Pattern and Simulation, Institute of Geographic Sciences and Natural Resources Research, Chinese Academy of Sciences, 11 A Datun Road, Beijing, 100101 People’s Republic of China; 2 College of Resources and Environment, University of Chinese Academy of Sciences, Beijing, China

**Keywords:** Social capital, Income disparities, Depressive symptoms, Left-behind children, China

## Abstract

**Background:**

Few prior studies have investigated the income gradient in child mental health from a socio-environmental perspective. In an age when child mental health problems in a rapidly changing social environment have become a worldwide issue, an understanding of the socio-environmental mechanisms of the income disparities in child mental health outcomes is imperative and cost-effective.

**Methods:**

By conducting structural equation analyses with Chinese nationally representative survey data, this study explored the family income gradient in child depression and its potential socio-environmental pathways at the neighborhood, family and school levels, differentiating left-behind and not-left-behind children.

**Results:**

We found a robust family income gradient in depressive symptoms. Neighborhood cohesion mitigated the income gradient in depressive symptoms by playing a suppression role. School social capital acted as a mediator. Neighborhood trust, neighborhood safety and family social capital played no significant impact. The mitigating and mediating roles of social capital components were significant among only the not-left-behind children.

**Conclusions:**

To reduce income-related inequalities in child mental health in the long run, integrating policies that directly reduce poverty with policies that improve distal socio-environments is necessary.

**Supplementary Information:**

The online version contains supplementary material available at 10.1186/s12889-023-16264-9.

## Background

The income gradient in health, a phenomenon that wealthier people tend to have better health outcomes throughout the income distribution, has its antecedents in childhood [1]. At a critical stage for acquiring cognitive and social-emotional skills, childhood circumstances have long-term consequences on subsequent adulthood experiences. Because there may exist a vicious cycle of poor living conditions, poor health in childhood, poorer cognitive performance, lower educational attainment, poorer-paid jobs, reduced inputs in health, and deteriorated adult health [2], a sound understanding of the income gradient in child health not only contributes to improving child health, but also holds long-term implications for improving the life trajectories of socioeconomically disadvantaged people who currently have rather restricted social mobilities.

A complex of social and economic changes, such as the accelerated life pace, the rising anxiety in families, a more highly pressurized school culture and the penetration of cyberbullying everywhere, has contributed to increased mental health problems among children [3]. Globally, approximately 10% of children and adolescents are experiencing a mental disorder [4]. Since a broader trend of growing disparity in living conditions, the dramatic increase in single-headed households, the reduced parenting due to longer working hours, the more unpredictable working patterns and increased residential segregation more heavily affect disadvantaged children [5], a general pattern of a rising gap in child mental health is also reported [6]. Moreover, the inequality in child mental health has been argued to be further exacerbated by the ongoing COVID-19 pandemic which has pushed more families into poverty [7]. Because the cognitive and social-emotional skills acquired during childhood are critical in shaping future mental health and in assuming subsequent adult roles in society [8], and family income has been one of the strongest predictors of mental health problems among children [9], examining and understanding the family income gradient in child mental health are urgent and necessary.

Current research concerning the family income gradient in child health has been mainly working on physical health outcomes, and access to structural and material resources has been proposed as the primary interpretation. There are established family income gradient in child physical health outcomes such as self-reported health, chronic conditions, height, and weight [1, 10, 11]. The quality, quantity, and accessibility of material resources, including routine medical care, health education, nutrition intake, leisure time activities, housing conditions [1, 10], etc., are most frequently redeemed as the mechanisms. Although theoretically, socio-environmental components are potential mediators and psychosocial and structural factors are also important pathways [8], empirical studies incorporating these are limited. More studies are needed to delve into the family income gradient in child mental health and the socioenvironmental mechanisms that contribute to it.

There are close connections between family income and social environments, and between socioenvironmental components and child mental health. On one hand, social environmental components, such as cohesion, trust, familial interactions, support and networks, are closely associated with family income [11]. On the other hand, the quality of the distal environments where children grow up shapes their well-being. For example, early exposure to negative experiences in neighborhoods, schools or families, such as annoying neighbors, crime [12], poor teacher-student relationships, prolonged separation, all kinds of abuse and poverty [13], increases the risk of mental illness. Therefore, a poor family in a poor neighborhood with poor social capital is subjected to “multiple jeopardies” [14]. Because a great number of people can benefit from environmental interventions conducted at the population level even if in some cases its effect may be small, to improve child mental health and reduce inequalities, exploring the potential socioenvironmental pathways of the family income gradient in child mental health is necessary.

China’s rapid socioeconomic development and tremendous social changes have been accompanied by a rising prevalence and inequalities of child depression. The prevalence of nonsuicidal self-harm among Chinese children aged 13–18 is 27.4%, compared to 19.5% worldwide [15]. Contemporary children are living in changing social environments with the traditional neighborly relationship based on extended family networks being gradually replaced by a modern interpersonal connection based on regulations. Moreover, because of China’s strict household registration system and institutional barriers, there are tremendous numbers of left-behind children (LBC), both in the urban and rural areas, who are left behind in their home of origin by their parents seeking job opportunities outside of hometown [16]. Primarily due to their prolonged separation from migrant parents, compared to not-left-behind children (NLBC), LBC are subject to attenuated emotional attachment and confronted with more difficulties with social support and security. Moreover, according to previous empirical studies, LBC are more vulnerable to environmental and social disadvantages and prone to higher rates of mental health disorders [17]. It is highly possible that the socioenvironmental mechanisms of family income gradient in mental health vary between LBC and NLBC. As such, exploring the distal social environmental explanations of the income gradient in child depression, differentiating between LBC and NLBC, will extend disciplinary knowledge and provide insights into more effectively tackling child mental health issues.

### The family income gradient in child mental health

Ever since Case et al.’s (2002) seminal work on family income gradient in child health was published, extensive literature has emerged and there are firmly established income gradients in child general and chronic health conditions [9, 10, 18]. Comparatively, there is a smaller body of evidence on the income gradient in child mental health outcomes [19]. Although family income is regarded as an effective measure of absolute poverty, a “best-fit” index in exploring the socioeconomic correlates of child psychopathology, and an independent cause of disparities in child mental health [20], studies on the socioeconomic determinants of child mental health attest primarily to the impact of a composite measure of socioeconomic status, e.g., poverty, parental education, occupation and housing [21], family expenditures and consumption [22], or respondents’ assessment of ordinal economic status [23]. Moreover, indicators of child mental health conditions are dominantly based on proxy reports by parents or teachers [19], which were argued to be poor measures of endogenous traits among young children [9]. An investigation of mental health outcomes from children’s perspective is notably absent. Thus, more empirical studies are needed to delve into the family income gradient in self-reported child mental health outcomes.

### Social capital as the measurement of social environment and a potential mediator linking income to child health

Entering the mainstream of public health since the 1990s, social capital is one of the “essentially contested concepts” in social science research. Although a standardized measure is still lacking, the emphasis of social capital as social relationships between groups of people is common, and indicators such as trust, participation, cohesion, social norms and collective efficacy are frequently used [24, 25]. Social capital has been widely adopted as a primary measure of social environments in social science research [14]. Numerous studies addressing socioeconomic disparities in adult health from a socioenvironmental perspective employed indicators of neighborhood social capital as key predictors [14, 25, 26]. In addition, studies working on the socioenvironmental determinants of child wellbeing have covered social capital at the neighborhood, family and school levels [27, 28]. By promoting the spread of health-related norms and information and the utilization of health facilities and services, providing material resources, as well as fostering emotional support [14, 24], social capital has been an important protective factor for health, although mixed findings existed [24].

The levels of social capital components are closely related to the indicators of economic development such as household income. Because a lack of economic resources may restrain people’s participation in social activities, surfaces of contact and membership in social groupings [29], economic hardships have been widely reported to be closely related to a lower level of social capital such as limited networks, low social support, trust and cohesion [25, 29]. For children, social capital at the neighborhood, school and family levels, all of which are their primary places of activity, were argued to be closely related to their levels of family income [30]. According to studies from the Western context, children with low family income are more likely to live in neighborhoods with poor physical and social environments, which normally lack sufficient social support and a sense of trust between neighbors [24, 29]. Children from poor households are more likely to study in schools with poor conditions. And they are more likely to report poor relationships with teachers and peers, which are predictive of negative development [31]. Household economic insecurity may exert household members extra pressure and restain the informal control of familial process, and therefore jeopardize the positive interactions and relationships between parents and children [30].

In linking social environments to child health, three models, i.e., the institutional resources model, the relationships model, and the norms/collective efficacy model, have previously been put forward [11], which are theoretically very useful in understanding the underlying mechanisms of the family income gradient in child depression. The institutional resources model underscores personal factors and redeems people’s differential access to health-related material resources as a core channel. The two other models stress the crucial role of the distal social environment. Given that lower family income is normally linked to unfavorable social environments, the norms and collective efficacy model states that disadvantaged neighborhoods negatively influence neighborhood social norms and residents’ willingness to intervene for the common good, which may be detrimental to child health. Comparatively, the relationships model focuses on psycho-social pathways and emphasizes the role of the family environment in mediating the association between income and health.

Empirical studies on income gradient and child health have provided support for the institutional model, and the mediating roles of health shocks, nutrition, housing conditions and healthcare utilization have been reported [8]. Parenting style and family relationships have also been occasionally proposed as potential mediators [32]. Comparatively, although both the norms/collective efficacy and the relationships models posit the crucial role of distal social environments, there have been few empirical studies dealing with this. Extant studies have mainly explored the role of neighborhood social capital in linking neighborhood disadvantage to child wellbeing [11], the direct effects of multidimensional social capital, including social capital at neighborhood, school and family levels, on child health [27], or the potential role of family social capital in mediating the effect of poverty on child wellbeing [33]. As such, empirical studies investigating the role of the distal social environments in the income gradient in child health are needed.

### Mental health issues, social capital and left-behind children in China

Child mental health problems in China have become a national health issue. The rapid socioeconomic development has been accompanied by tremendous changes in the social and family structure, rapid urbanization, widened discrepancies in development and increased social competition. China’s relaxed family-planning policy has failed to reverse its downward trend of fertility rate. Children nowadays have been seen as the “only hope” of their families, and thus been subjected to high academic requirements, multiple family expectations, and increased psychological pressure [34]. Moreover, children are facing increasing stress and competition at school [35]. Mental health conditions have been a major burden of child diseases [15]. According to the latest national data, at least 30 million under 17 years children are struggling with behavioral or emotional problems, and approximately one in four adolescents report mild or severe depression [35].

Simultaneously, the distal social environment within which children grow up has also changed. The traditional neighborly relationship is attenuating, and a modern social capital characterized by cooperation and extensive trust is gradually emerging. Unlike the self-initiated and voluntary neighborhood in the Western discourse, the neighborhood in China is more of a grassroots unit of administration. On one hand, China’s former housing policies that integrated work with home have generated unique neighborhoods, with people living in the previous work unit houses exhibiting strong social ties regardless of socioeconomic status [26]. On the other hand, in contrast to the economically better-off urban neighborhoods, where residents normally share less and a sense of community is currently being cultivated, rural neighborhoods are more likely to have well-connected social networks with a rather slow flow of population [14]. Combining this, it has been reported that residents from old and socioeconomically disadvantaged neighborhoods have higher levels of cohesion and tend to be more closely bonded [36]. Given the dramatically different institutional conditions under which the Chinese neighborhood emerges and functions, the role of neighborhood social capital in the income gradients in child health may differ substantially from those of the Western contexts.

Primarily because of institutional barriers, the majority of China’s massive floating population, who migrate to urban areas in search of better job opportunities, have to leave their children at home in the care of others, creating numerous LBC. Historically, LBC refers to mainly left-behind children living in rural areas. With the structural change in China’s migration populations, i.e., the massive rise in the number of urban-to-urban migrants, LBC living in urban areas has dramatically increased in the past decade [37]. According to the latest national data, compared to that of 2010, in 2020, the total number of rural LBC children was 41.77 million, which had increased by 5.2%; in comparison, the total number of urban LBC had reached 25.16 million, which had increased by 65.4% [16]. Compared to NLBC, LBC may suffer from inadequate parental supervision, less emotional care and support, increased workloads, more barriers to communication, and unmet needs for parental affection [38]. Due to a lack of parent–child interactions and parental tutoring, LBC may be unable to gain timely attention and support from their teachers and are less likely to be involved in neighborhood activities [39]. Although parental migration to major cities may yield large remittances for households and therefore benefit LBC, the adverse situation created by parent–child separation could nevertheless generate adverse effects [40]. According to the most recent research and report involving urban LBC, with one or both parents working away from home had generated similar adverse effects on the mental health of both urban and rural LBC [16, 41]. Given the varying social processes they undergo, differentiating between LBC and NLBC in studying the socio-environmental mechanisms of the income gradient in child mental health will provide greater insights.

### The present study

**Fig. 1:**
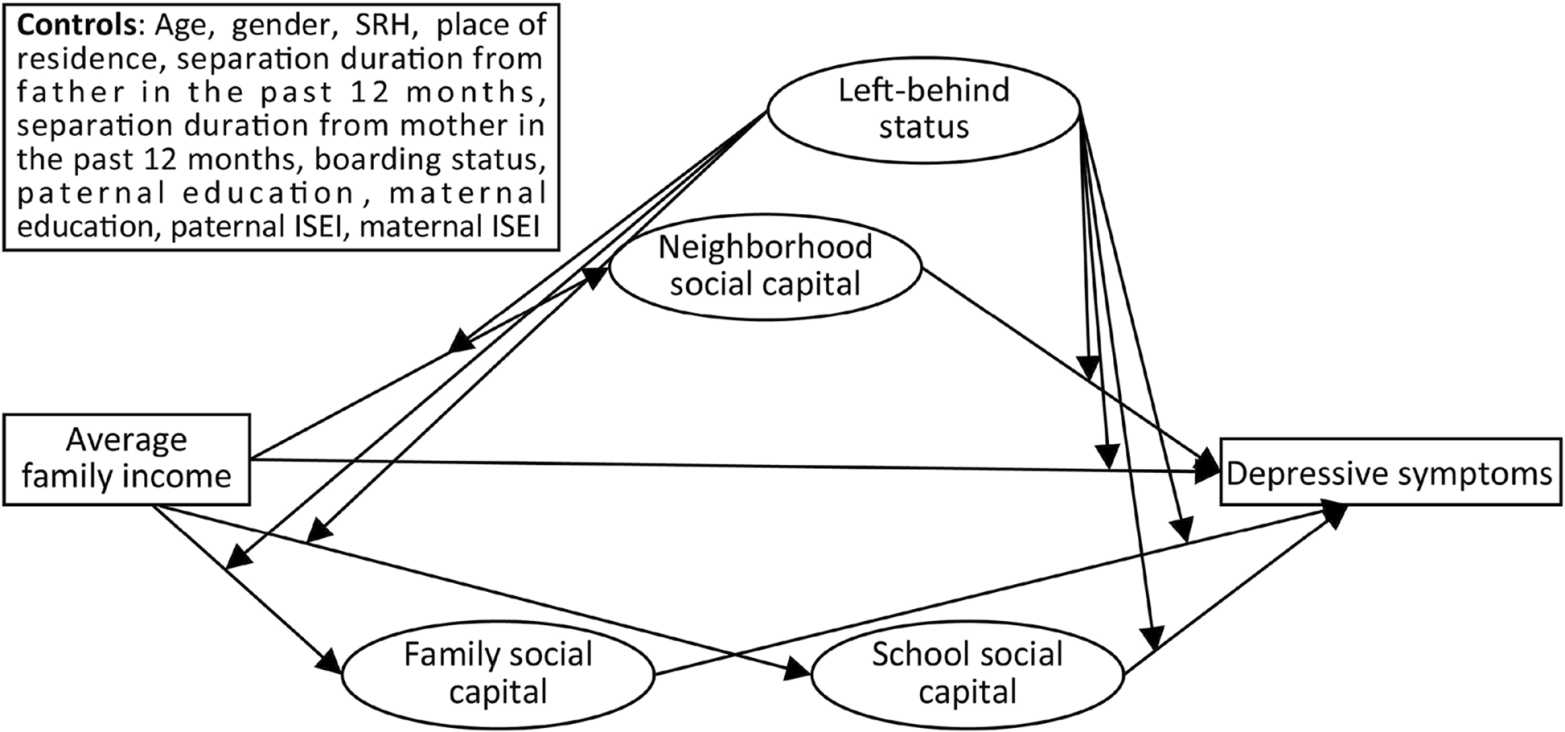
Conceptual framework

More empirical studies on the income gradient in self-reported child mental health are needed. Moreover, there is a lack of evidence supporting the norms/efficacy model from a socioenvironmental perspective. Applying structural equation modeling to nationally representative data, this study seeks an in-depth understanding of the potential socioenvironmental pathways in the family income gradient in child mental health. Considering the cultivation of Chinese social capital, the presence of depression as the leading issue, and data availability, indicators of depressive symptoms are used to reflect mental health, and indicators of neighborhood, family and school social capital are used to measure contextual social environments. We first investigate whether there is a family income gradient in depression. We then explore whether indicators of neighborhood, family and school social capital play any mediating role. We finally stratify our analyses by LBC and NLBC (Fig. 1). Specifically, we hypothesize:

- (1) There is a significant and robust family income gradient in child depression.
- (2) Components of neighborhood, family and school social capital mediate the association between family income and depression.
- (3) Left-behind status plays a moderating role in the socioenvironmental mechanisms of the income gradient in child depression.

## Methods

### Data source

China Family Panel Studies (CFPS), a nationally representative survey, which has been conducted every two years since 2010 on the economy, society, population and health, provides the original data source. CFPS is hierarchically designed to collect individual, household, and neighborhood data. The individual child database collects information on respondents 16 years or younger, while respondents 17 years or older answer individual adult questionnaires. The family database provides familial demographic and socioeconomic characteristics, and the neighborhood database focuses mainly on neighborhood social welfare, population, facilities and economic information. Starting with the CFPS 2012 follow-up, proxy questionnaires were used to collect the basic information of household members who were absent during the interview. CFPS 2016 began using the same questionnaire for a mixed model of face-to-face interviews and computer-assisted telephone interviews (CATI), which enabled a collection of subjective information of family members who were not physically present at the time of the interview. CFPS was approved and monitored by the Biomedical Research Ethics Review Committee of Peking University (IRB00001052-14010, Beijing, China). Representing 95% of the Chinese population and covering 25 of the 31 mainland provinces, the CFPS records and maintains comprehensive and high-quality data.

In this study, the child database provided children’s basic information, the family database provided family member relationships and familial socioeconomic information, the neighborhood database provided residential information, and the adult database provided parental characteristics and residents’ perceptions of neighborhood social environments. The CFPS questionnaires concerning neighborhood social capital indicators across waves were not identical. For example, CFPS 2018 merely recorded information on trust, and the newly and publicly issued CFPS 2020 had not yet provided neighborhood-related information; in contrast, CFPS 2016 had a rather comprehensive record on trust, cohesion and safety. In this case, the cross-sectional data of CFPS 2016 were selected in this study. Because the CFPS collected children’s perceptions of school social environment and the primary guardians’ involvement in children’s daily lives only among children aged 10–15 years, to enable a synthetic analysis, children of this age group were focused. We used paternal ID, maternal ID, family ID, and neighborhood ID to link databases. The first-round match yielded a sample size of 2 486 children. To eliminate the potential confounding of parental marital status, 173 observations from single-parent families and 9 with deceased parent(s) were excluded. Eventually, 2 304 children were included.

### Left-behind and not-left-behind children

There is a lack of a universal standard for the definition of LBC. Rural children aged 16 or younger with one or both parent(s) working and living in another city were previously regarded as LBC in 2016 [42]. With the structural change in China’s internal migration patterns, which is characterized by the magnificent increase in urban-to-urban population, there is an ever-growing population of urban LBC [41]. According to the latest document of the National Bureau of Statistics of China and United Nations International Children’s Emergency Fund (UNICEF) China in 2023, LBC includes both rural and urban LBC which refers to children aged 0–17 years who live in location of their household registration, and who have one parent or both parents migrating outside of hometown for six months or longer [16]. Empirically, albeit at least 5 months’ separation [43] or 7 months’ separation [38] were occasionally applied, 6 months’ separation in the past 12 months from one or both migrating parent(s) was most frequently used as the threshold [37, 40]. To keep in line with official definition and common practice, and to take into account the ongoing increase in urban LBC in contemporary China, LBC in this study included both urban and rural children who separated from one parent or both parent(s) for at least 6 months. Accordingly, 559 of the 2 304 children were LBC, and 1 745 were NLBC.

### Measures

#### Depressive symptoms

The Center for Epidemiological Studies Depression Scale for Children (CES-DC), a 20-item self-report depression tool, was used to measure depression. CES-DC is one of the most widely used self-reported inventories for the assessment of depression or negative mental health among children [27]. CES-DC ranges from 0 to 60 with higher scores indicating a higher possibility of depression (Supplementary Table A.1).

#### Family income

Following extant studies [1, 44], we used the annual household income to measure family income. Annual household income was constructed based on the total calculation of five primary components, i.e., wage, total business income, property income, transfer income and other income. Given the potential underestimation of family income in less commercialized regions due to the omission of self-consumed products, regional variations in living expenses were adjusted, and the total income of agricultural production was converted, adjusted and included in family income.

#### Neighborhood, family and school social capital

*Neighborhood cohesion, trust and safety.* There is neither a unified definition nor a standard measure. Previous studies have primarily used members’ overall satisfaction and subjective assessments of neighborhood upkeep, shared values, trust, neighborly relationships, or safety to signify various dimensions of neighborhood social capital [24]. Following extant practice, neighborhood social capital was measured by aggregating the evaluations of within-neighborhood members. Considering data availability, indicators concerning safety, trust and cohesion, which underscored various dimensions of neighborhood social capital, were used (Supplementary Table A.2). Answers were provided by all the respondents interviewed belonging to that neighborhood. Eventually, 12 344 adult respondents from 709 neighborhoods, with an average of more than 17 respondents per neighborhood (range = [8–83]), were involved.

*Family social capital.* Although the concept of family social capital was introduced by Coleman as early as the 1980s [45], it lacks a universal measure. Components of family social capital, such as family ties, parent monitoring, parent–child interactions and parental involvement [24], have been variably constructed. Drawing heavily from Coleman’s postulation of family social capital as the attention that adults give to children and the intra-family relationships, 6 indicators concerning the involvement of the primary guardian in a child’s daily life were used. A total of 2 304 primary caregivers responded to questions about how frequently they “gave up watching TV shows to avoid disturbing child”, “discussed what happened at school with child”, “asked the child to finish homework”, “checked the child’s homework”, “restricted the child from watching TV” and “knew with whom the child was when he or she was not at home”. Responses scored from 1 to 5, with 1 meaning “never” and 5 meaning “very often”.

*School social capital.* Children’s perceptions of school climate, which involve multiple dimensions of teacher-student relationships, trust, satisfaction and connectedness, are commonly used to indicate school social capital, although measures vary [30, 46]. Referencing extant studies and the data, children’s feelings about their school atmosphere, i.e., their perceptions of satisfaction with their “school”, “class adviser”, “Chinese language teacher”, “math teacher”, and “foreign language teacher” were used. Response scored from 1 to 5, with 1 being “extremely unsatisfied” and 5 being “extremely satisfied”.

#### Covariates

Age, gender, self-reported health (SRH), place of residence, living arrangements, boarding status, paternal and maternal education, and paternal and maternal International Standard Classification of Occupation (ISEI) were included. The educational attainment of children was excluded because it was highly correlated with age.

### Analytical strategy

To assess the suitability of aggregating individual perceptions as neighborhood social capital measures, the r_wg_ statistic was used to examine within-neighborhood homogeneity. An r_wg_ value of 0.7 or higher indicated high within-group homogeneity [47].

Cronbach’s α was applied to test the internal consistency reliability. Structural equation modeling (SEM) was applied to examine structural relationships. First, we used the structural model to test the association between income and depression. Then, we used confirmatory factor analyses to evaluate measurement models of four latent variables (i.e., neighborhood cohesion and trust, family social capital and school social capital). Furthermore, we used Bootstrapping methods to investigate whether social capital mediated the relationship between income and depression. Finally, we used multi-group structural analyses, which included a set of 8 models, to examine whether left-behind status played a moderating role. Model 1 was the fully constrained model with all the factor loadings and coefficients being equal across LBC and NLBC. The factor invariance of the latent construct of neighborhood trust and cohesion, family social capital and school social capital was tested in Model 2. To reveal whether the mediating effects found in the mediation analyses were generalizable across groups, the paths linking income to depression, income to neighborhood cohesion, neighborhood cohesion to depression, income to school social capital, and school social capital to depression were released in Models 3 to 7, respectively. In Model 8, paths exhibiting significant differences were released together to test the robustness and find the best model.

Multiple indices were considered to evaluate fitness. (1) The chi-square (χ^2^), where an associated probability value indicating non-significant χ^2^ stands for a close fit.^1^ (2) The Root Mean Square Error of Approximation (RMSEA), where values less than 0.08 indicate an acceptable fit. (3) The Comparative Fit Index (CFI), where values larger than 0.9 indicate a good fit. (4) Tucker Lewis index (TLI), where values higher than 0.9 indicate fitting well. (5) Standardized Root Mean Square Residual (SRMR), where values lower than 0.08 indicate an acceptable fit.

### Missing data

Except for residence, gender, CES-DC, age and SRH, the majority of variables had missing values. The proportions ranged from 0.48% for separation duration from mother to 13.5% for maternal ISEI. Preliminary analyses implied that missing values were not missing completely at random (MCAR) (e.g., residence and maternal education were independently predictive of missing maternal ISEI). Given the estimation bias of listwise or pairwise deletion methods when data were not MCAR, Bayesian imputation, which conducted multiple imputations based on the posterior predictive distribution of missing values, was applied. We first used Bayesian imputation to generate 20 single and equally plausible datasets. Then, we conducted a posterior simulation with each of the 20 complete datasets using standard structural equation techniques. Finally, using Rubin’s rules for scalar estimands [48], we combined the 20 sets of results to produce one set of estimates. The *norm2* package of the R software was used to conduct imputation and estimation.

## Results

### Descriptive statistics

There was no significant difference between LBC and NLBC in the mean scores of CES-DC, family income, gender, SRH, age, parental ISEI, neighborhood trust, safety and school social capital. Urban areas had significantly more NLBC. 55% were males, which was comparable to the sex ratio at birth (111.3) in the latest national census. 44% of LBC were boarding, significantly higher than NLBC (27%). Months living with parents were significantly shorter among LBC (2.86 vs. 9.5, 5.54 vs. 10.21), parental education was significantly lower among LBC (6.93 vs. 7.84, 5.32 vs. 6.76), neighborhood cohesion was higher among LBC (0.48 vs. 0.4), and indicators of parental involvement were significantly higher among NLBC (Table 1).

**Table 1:** Descriptive statistics of the total, left-behind and not-left-behind children (Mean (SD))

	Total	LBC	NLBC
CES-DC	10.08(5.88)	10.21(5.99)	10.04(5.59)
Family income per capita (yuan)	9945 (11728)	9447 (10893)	10144 (12038)
Residence (Urban, %)***^a^	41	32	44
Gender (Male, %)	55	53	55
Boarding (Yes, %)***^b^	31	44	27
SRH	3.97(0.94)	3.92(0.97)	3.98(0.93)
Age (Years)	12.37(1.68)	12.46(1.68)	12.34(1.68)
Months living with father***^c^	7.89(4.64)	2.86(2.92)	9.50(3.87)
Months living with mother***^d^	9.08(4.32)	5.54(4.77)	10.21(3.47)
Paternal ISEI	32.97(13.44)	32.81(12.04)	33.02(13.85)
Maternal ISEI	32.32(12.95)	32.38(11.99)	32.30(13.26)
Paternal education (Year)***^e^	7.63(4.14)	6.93(4.17)	7.84(4.11)
Maternal education (Years)***^f^	6.42(4.54)	5.32(4.45)	6.76(4.52)
Neighborhood cohesion***^g^	0.42(0.47)	0.48(0.45)	0.40(0.47)
Neighborhood trust	0.10(0.44)	0.12(0.44)	0.09(0.45)
Neighborhood safety	0.15(0.46)	0.15(0.45)	0.15(0.47)
Satisfaction with school	4.04(0.95)	4.01(0.96)	4.05(0.96)
Satisfaction with class adviser	4.29(0.96)	4.31(0.95)	4.29(0.97)
Satisfaction with Chinese language teacher	4.25(0.95)	4.27(0.93)	4.24(0.97)
Satisfaction with Math teacher	4.19(0.10)	4.21(0.99)	4.19(1.00)
Satisfaction with Foreign language teacher	4.07(1.02)	4.08(1.00)	4.07(1.02)
Give up watching TV shows to avoid disturbing child ***^h^	3.42(1.29)	3.28(1.35)	3.48(1.27)
Discuss what happens at school with child***^i^	3.18(1.16)	2.99(1.21)	3.25(1.13)
Ask the child to finish homework***^j^	3.96(1.04)	3.84(1.10)	4.00(1.01)
Check the child's homework***^k^	3.01(1.37)	2.76(1.41)	3.10(1.35)
Restrict the child from watching TV	3.43(1.15)	3.36(1.19)	3.45(1.13)
Know with whom the child is when he/she is not at home	2.65(1.35)	2.61(1.39)	2.67(1.33)

Note: ^a^ χ^2^(1,N = 1) = 27.96, *P* < .001

^b^ χ^2^(1,N = 1) = 60.73, *P* < .001

^c^ χ^2^(1,N = 1) = 1396.47, *P* < .001

^d^ χ^2^(1,N = 1) = 630.47, *P* < .001

^e^ χ^2^(1,N = 1) = 41.23, *P* < .001

^f^ χ^2^(1,N = 1) = 19.20, *P* < .001

^g^ χ^2^(1,N = 1) = 11.57, *P* = .001

^h^ χ^2^(1,N = 1) = 10.82, *P* = .001

^i^ χ^2^(1,N = 1) = 24.59, *P* < .001

^j^ χ^2^(1,N = 1) = 11.03, *P* = .001

^k^ χ^2^(1,N = 1) = 30.85, *P* < .001

### Test of the measurement models

**Fig. 2:**
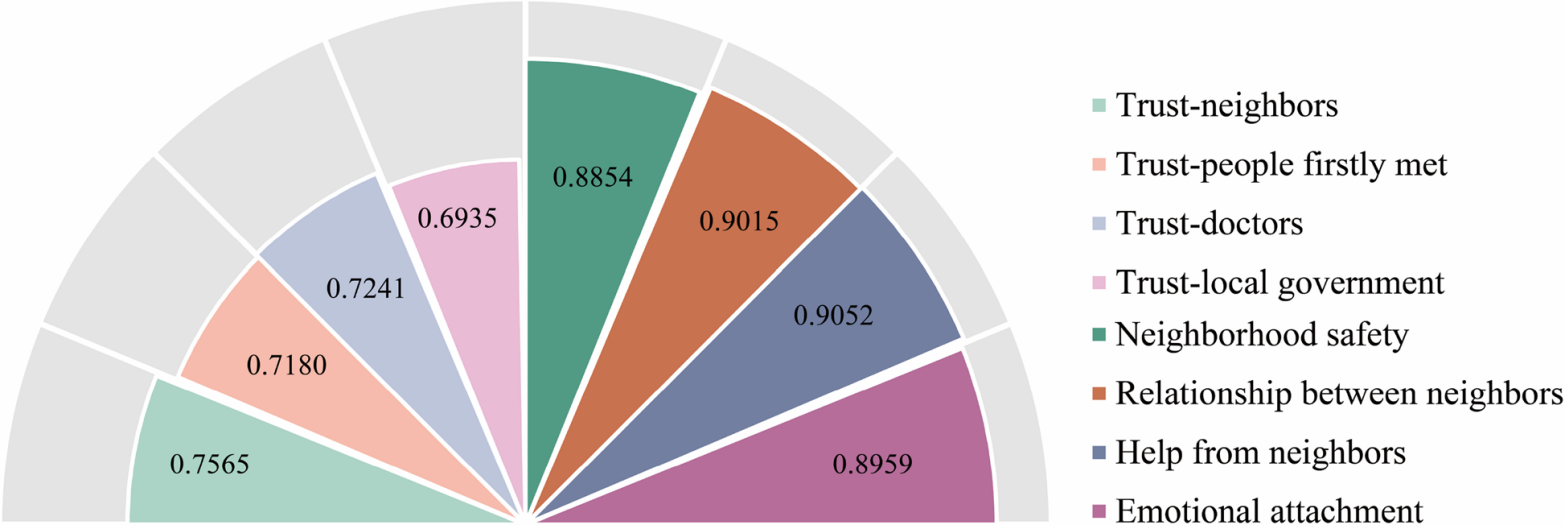
Within-neighborhood Interrater Reliability Coefficients of indicators of neighborhood trust, safety and cohesion

The r_wg_s for neighborhood social capital components were all approximately or higher than 0.7 (Fig. 2), implying the reasonability of aggregating individual data. Cronbach’s α for neighborhood cohesion, trust, family and school social capital exhibited acceptable values (0.697, 0.689, 0.681 and 0.791).^2^ Additionally, Friedman chi-square tests for all latent constructs were significant, indicating significant discrepancies in answers. The measurement models indicated a good fit. For neighborhood cohesion and trust: Chi-square: 72.14 (df = 13; *p* < 0.001), RMSEA = 0.040, CFI = 0.984, TLI = 0.965, SRMR:0.019. For family and school social capital: Chi-square: 64.26 (df = 38; *p* = 0.005), RMSEA = 0.017, CFI = 0.995, TLI = 0.992, SRMR:0.034. Observed variables were all significantly loaded on their latent constructs, and the standard factor loadings ranged from 0.38 to 0.78 (Table 2), all higher than the threshold of 0.3 [49].

**Table 2:** Standardized factor loadings of observed variables on latent constructs

Latent construct	Observed variable	Factor loading
Neighborhood cohesion	Relationship between neighbors	0.64
	Help from neighbors	0.59
	Emotional attachment to neighborhood	0.70
Neighborhood trust	Neighbors	0.58
	People meet for the first time	0.45
	Local government official	0.55
	Doctors	0.38
Family social capital	Give up watching TV shows to avoid disturbing child	0.45
	Discuss what happens at school with child	0.54
	Ask the child to finish homework	0.45
	Check the child's homework	0.66
	Restrict the child from watching TV	0.40
	Know with whom the child is when he/she is not at home	0.38
School social capital	School	0.57
	Class adviser	0.78
	Chinese language teacher	0.75
	Math teacher	0.66
	Foreign language teacher	0.55

### Test of structural models: the mediating effect of social capital

**Fig. 3:**
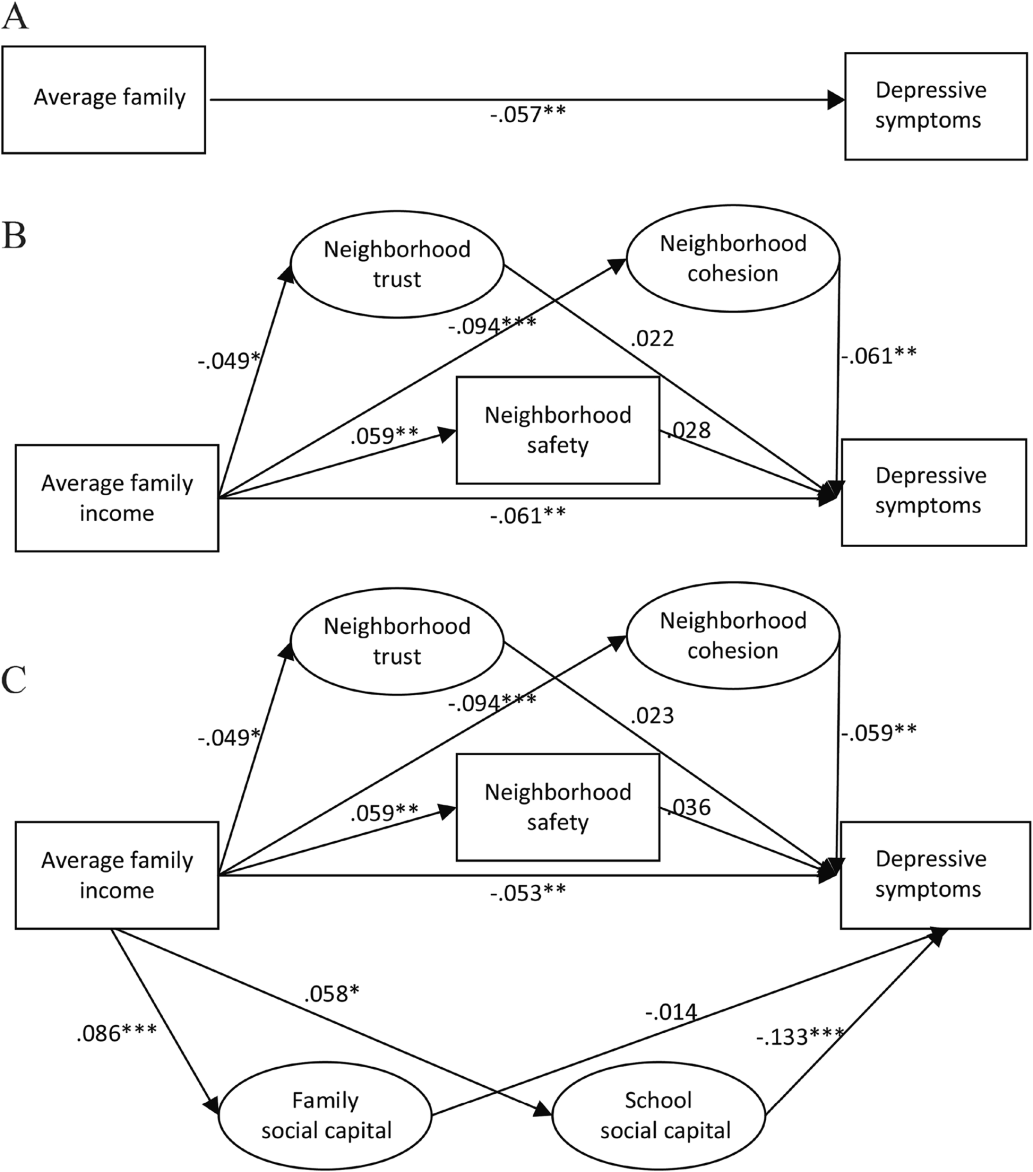
Standardized estimates with social capital as potential mediators. Notes: Average family income was logarithm transformed. **A**: Chi-square: 115.61 (df = 29; *p* < 0.001), RMSEA:0.036, CFI:0.969, TLI:0.941, SRMR:0.041. **B**: Chi-square: 214.36 (df = 50; *p* < 0.001), RMSEA:0.038, CFI:0.948, TLI:0.906, SRMR:0.040. **C**: Chi-square: 802.37 (df = 238; *p* < 0.001), RMSEA:0.032, CFI:0.934, TLI:0.916, SRMR:0.045

The goodness-of-fit indices demonstrated satisfying results (Fig. 3). There was a significant income gradient in depression, with higher income predicting less depression (Fig. 3(A), β = -0.057, *p* < 0.01). After neighborhood trust, cohesion and safety were included (Fig. 3(B)), the income gradient in depression persisted (β = -0.061, *p* < 0.01). Income had a positive direct effect on safety (β = 0.059, *p* < 0.01) and negative direct effects on trust (β = -0.049, *p* < 0.05) and cohesion (β = -0.094, *p* < 0.001). Higher neighborhood cohesion was linked to less depression (β = -0.061, *p* < 0.01). Trust and safety showed no significant impact. Bootstrap mediation analyses indicated a significant indirect effect of income on depression through cohesion (β = 0.006, *p* < 0.05) (Table 3).

**Table 3:**
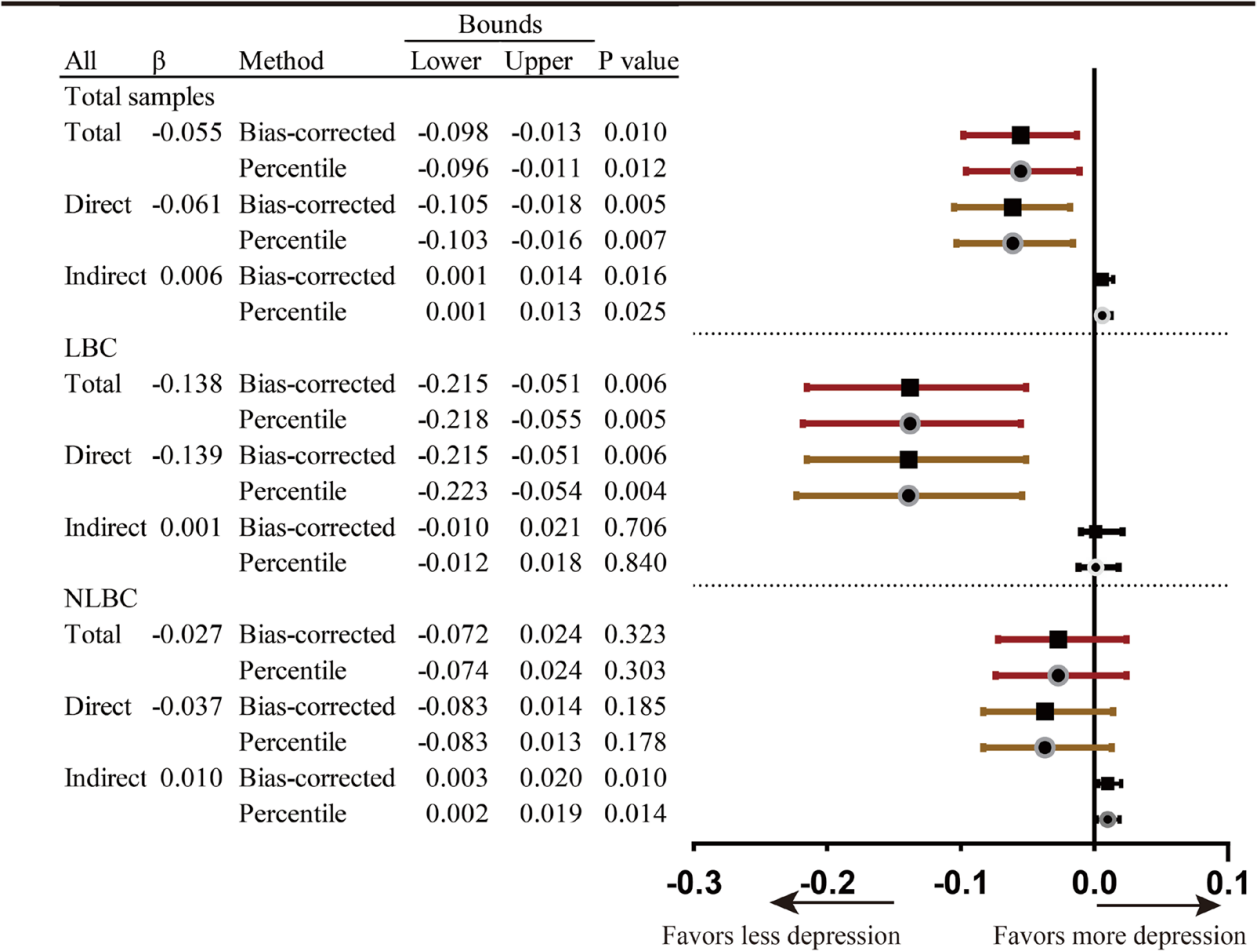
Bootstrap analyses of neighborhood cohesion as potential mediator linking family income to children’s depressive symptoms (Standardized)

Notes: Red error bars-total effect, brown error bars-direct effect, black error bars-indirect effect; error bars with square symbols-Bootstrap confidence with percentile method, error bars with circle symbols- Bootstrap confidence with Bias-corrected percentile method

After family and school social capital were included, the significant impact of income (β = -0.053, *p* < 0.01) and cohesion (β = -0.059, *p* < 0.01) on depression, and income on cohesion (β = -0.094, *p* < 0.001) persisted (Fig. 3(C)). Income had a significant direct effect on family (β = 0.086, p < 0.001) and school social capital (β = 0.058, *p* < 0.05), and school social capital had a significant direct effect on depression (β = -0.133, *p* < 0.001). Bootstrap analysis implied a significant indirect effect of income on depression through school social capital (β = -0.006, *p* < 0.05) (Table 4).

**Table 4:**
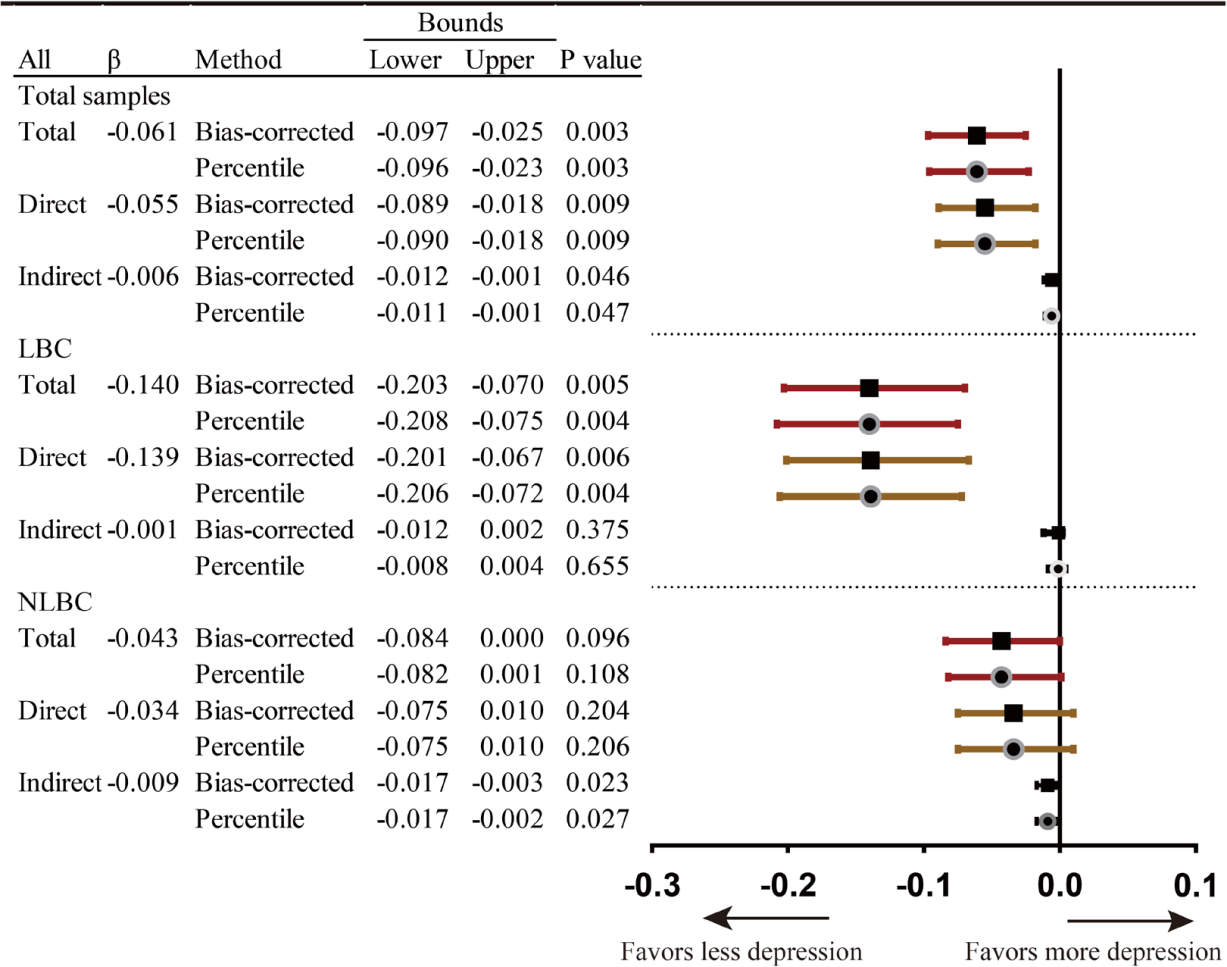
Bootstrap analysis of school social capital as potential mediator linking family income to children’s depressive symptoms (Standardized estimates)

Notes: Red error bars-total effect, brown error bars-direct effect, black error bars-indirect effect; error bars with square symbols-Bootstrap confidence with percentile method, error bars with circle symbols- Bootstrap confidence with Bias-corrected percentile method

### Test of structural models: the moderating effect of left-behind status

Table 5 lists the comparison of multiple alternative models. From Model 1 to 2, the decrease in χ^2^ was not significant, implying that all the factor loadings were equal between groups. Therefore, the measurement invariance of latent variables was established, and a test of structural invariance could be subsequently conducted. The significant decrease in χ^2^ in Models 3, 4 and 7 implied that the hypotheses that the paths from family income to depressive symptoms, from family income to neighborhood cohesion, and from school social capital to depressive symptoms were equal were false. In contrast, the insignificant decrease in χ^2^ in Models 5 and 6 signified that there was no significant difference in the paths from neighborhood cohesion to depressive symptoms and from family income to school social capital. With the simultaneous release of significantly different paths in Models 3, 4, and 7, Model 8 showed the best fit with the lowest χ^2^ value.

**Table 5:** Comparison of model fitness: multiple alternatives comparing left-behind children and not-left-behind children

Model	Model description	χ^2^	df	△χ^2^	△df	p
1	Full constrained model with all the factor loadings and paths being equal across LBC and NLBC	2148.332	563			
2	As model 1, release the factor loadings of neighborhood trust, neighborhood cohesion, family social capital and school social capital	2123.478	543	24.854	20	0.207
3	As model 1, release the path from family income to depressive symptoms	2144.523	562	3.809	1	0.051
4	As model 1, release the path from family income to neighborhood cohesion	2143.581	562	4.751	1	0.029
5	As model 1, release the path from neighborhood cohesion to depressive symptoms	2147.859	562	0.473	1	0.492
6	As model 1, release the path from family income to school social capital	2148.329	562	0.003	1	0.959
7	As model 1, release the path from school social capital to depressive symptoms	2144.124	562	4.208	1	0.040
8	As model 1, release the paths from family income to depressive symptoms, from family income to neighborhood cohesion, and from school social capital to depressive symptoms	2137.019	560	11.313	3	0.010

**Fig. 4:**
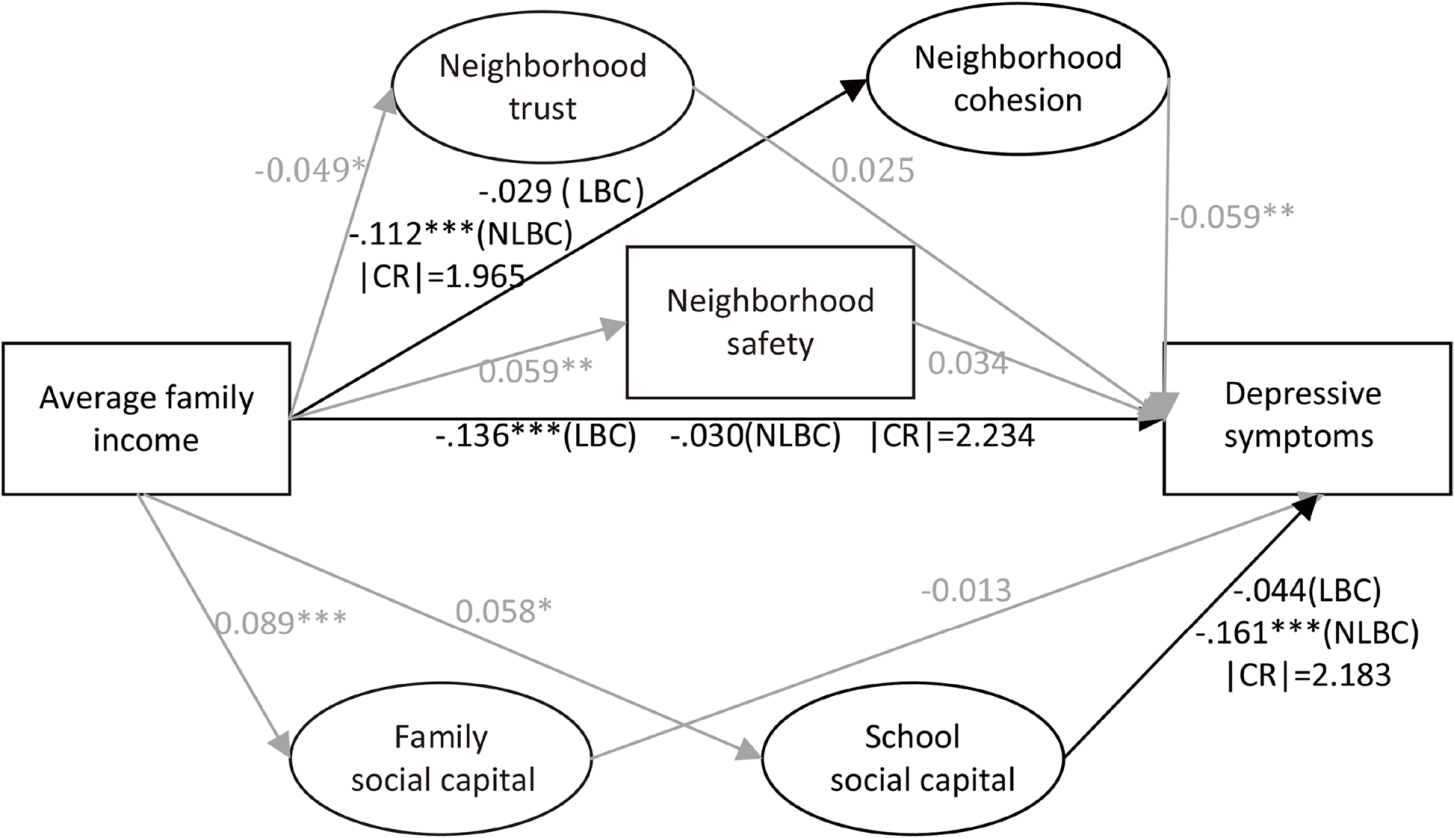
Standardized estimates of multi-group structural modeling. Notes: 1. CR: critical ratio. |CR|≥ 1.96, significant differences between parameters at 5%; |CR|≥ 2.58, significant differences between parameters at 1%; |CR|≥ 3.29, significant differences between parameters at 0.1%. 2. Model fit: Chi-square: 2137.019 (df = 560; *p* < 0.001), RMSEA:0.035, CFI:0.918, TLI:0.903, SRMR:0.068

Figure 4 shows the results of multi-group analyses. Income and cohesion were more closely linked (|CR|= 1.965) among NLBC (β = -0.112, *p* < 0.001) than LBC (β = -0.029, *p* = 0.481). The impact of school social capital on depressive symptoms was significantly (|CR|= 2.183) stronger among NLBC (β = -0.161, *p* < 0.001) than among LBC (β = -0.044, *p* = 0.35). In contrast, the association between income and psychological symptoms was significantly (|CR|= 2.234) stronger among LBC (β = -0.136, *p* = 0.001) than among NLBC (β = -0.030, *p* = 0.211). There were no significant differences in the association between cohesion and depressive symptoms or between income and school social capital. There was a significant suppression effect of neighborhood cohesion in the relationship between income and mental health among NLBC (see Table 3, total effect: β = -0.027, *p* > 0.05; indirect effect: β = 0.010, *p* < 0.05). Moreover, the significant mediation effect of school social capital was merely among the NLBC (see Table 4, NLBC, total effect: β = -0.043, *p* > 0.05; indirect effect: β = -0.009, *p* < 0.05) (see Fig. 4).

## Discussion

As hypothesized, our study revealed a robust income gradient in child depressive symptoms. The higher the family income was, the less depressed the children were. This finding verifies the social causation theory and the argument that socioeconomic status and child mental health are closely associated [50]. Extant studies on the income gradient in child health have focused mainly on physical outcomes, and surrogate indicators of income such as occupation, education and housing are frequently used [21], and parent- or teacher-reported mental health conditions are commonly adopted. Ours empirically examining the family income gradient in self-assessed depressive symptoms extends current research and expands our knowledge of the socioeconomic disparities in child mental health.

Our finding of the negative relationship between income and cohesion is opposite to that of the Western studies, which reported that more affluent families were more likely to live in neighborhoods with higher levels of social cohesion [51]. We propose the unique cultivation of Chinese neighborhoods as a reason. As the grassroots unit of administration [14], neighborhoods can be divided into three categories. The first is the rural village, where kinship and geographical relations are emphasized, the role of public service institutions is disregarded and economic conditions are not important in cultivating solidarity and support. The second is the “public housing neighborhood”, which was generated in the pre-reform era and formerly integrated residences, social welfare and workplaces. With the gradual implementation of housing reforms since the 1990s, welfare provision ended, and public housing was sold to tenants at discounted prices. However, a strong sense of neighboring among former residents has been preserved [26]. The third is the “commodity housing neighborhood”, which is a private housing complex with better facilities and higher prices. Because neighborhood-based social ties account for a small portion of social networks, there are few interactions and connections among residents in economically better-off commodity housing neighborhoods [36]. In this context, neighborhood cohesion in China is negatively associated with income. The protective role of cohesion on depression is not difficult to grasp and is in line with previous findings [39, 52]. Providing emotional support, transmitting healthy behaviors, seeking public resources and strengthening good public norms [53] are all possible channels through which cohesion improves child mental health.

Being negatively associated with income and protective against depression, our findings indicate that neighborhood cohesion plays a suppressive role in the family income gradient in child depression. The magnitude of the relationship between family income and child depression increased with the inclusion of neighborhood cohesion as an intermediary variable, which indicates neighborhood cohesion to be a suppressor variable according to extant definition [54]. Because family income exerted a negative direct effect on the occurrence of depression, and a positive indirect effect on the occurrence of depression through neighborhood cohesion, therefore, the total negative effect of family income on depression has been attenuated by neighborhood cohesion. In other words, different from previous work from the Western context which redeemed cohesion as a mediator linking economic status to health [11], ours indicates that neighborhood cohesion in the Chinese context has suppressed the family income gradient in child depression. This finding implies that since the level of neighborhood cohesion can be higher for poor families in the Chinese culture, improving cohesion is very promising in leveling the family income gradient in child depression.

Our multi-group analyses further revealed a suppressive role of neighborhood cohesion among only the NLBC. The varying conditions that LBC face may help explain this finding. Because parents function as gatekeepers to children’s neighborhood settings, the absence of parents could take away resources inherent in their social connections in the communal sphere [22]. Moreover, even with the same level of objective neighborhood cohesion, LBC exhibit a lack of enthusiasm for participating in community activities compared to NLBC [27]. Therefore, LBC may benefit less from the emotional support, good public norms and resources associated with neighborhood cohesion, and the role of neighborhood cohesion in suppressing the family income gradient in depression may be significant among only the NLBC.

We found that neighborhood safety played no significant mediating role. It is not difficult to understand the positive association between income and safety. However, although neighborhood safety was previously found to affect mental health [55], our study failed to find this. This may be due to two reasons. The first is the measurement of neighborhood safety. Previous studies used children’s perceptions of safety, and ours used adult respondents’ reports. Although aggregating adult members’ reports may be more reasonable and objective in assessing the security of a neighborhood, children might feel differently. Also, limited by data source, we used one general question “How do you rate public safety in your neighborhood?”. However, different components, such as property safety and personal safety, may impose varying effects [56]. The second is the robustness issue. Searching the literature, several studies reported no significant association between safety and either physical or mental health outcomes[57, 58]. As such, the role of neighborhood safety in mediating the income gradient in depression may be trivial.

Our study indicates school social capital to be a significant mediator. Lower income and lower school social capital is predictive of more depression. Children with lower family incomes are more likely to study in schools with poor conditions and a poor learning environment is closely linked to a higher risk of psychological distress. In addition, students with lower family incomes are more likely to report poor relationships with teachers and peers, which are predictive of negative development [31]. Our findings support the previous argument that school social environments are protective against child mental health problems [50].

Similar to that of neighborhood cohesion, the mediating role of school social capital was among only NLBC. Very few previous studies have examined the mediating role of school social environment on the income gradient in mental health among both LBC and NLBC. We propose two possible explanations. First, because of a lack of parent–child interactions and parental tutoring, compared to NLBC, LBC are less likely to acquire timely attention and responses from their teachers when they encounter difficulties, which results in insufficient school social capital. Second, due to parental absence, LBC are more likely to be boarding. Because boarding children experience school as the primary place of everyday life and tend to treat their teachers as authorities [27], it is possible that their true feelings were hidden. Our finding agrees with previous research arguing for the unremarkable effect of school social capital on the mental health of LBC [27].

We failed to find family social capital to be a significant mediator. Because family income significantly affects the way a child is raised and the resources that the family provides, the positive link between income and family social capital is understandable. Unlike previous findings indicating the beneficial impact of family social capital on child mental health [30, 46], we found no significant relationship. On reflection, we propose the mixed effects that family social capital may have in the Chinese context to be the reason. With education being the core channel for social mobility and educational achievement always being the top societal concern[41, 59], Chinese children nowadays bear multiple family expectations. Although family involvement in children’s daily lives can be beneficial to mental health and family investment in education is helpful in improving children’s development, over-involvement in daily life and education may go to extremes and generate a heavy burden. Thus, it is possible that the relationship between family social capital and child depression is not significant. Furthermore, the significant relationship between income and family social capital and the insignificant relationship between family social capital and child are very likely to cause an insignificant mediating effect.

Ours is one of the few studies that has empirically explored the family income gradient in child depression using nationally representative data. Our investigation of the socio-environmental pathways in the income gradient in child depression provides solid empirical support for the norm and collective efficacy model, and enriches the validity of the institutional resources model and the relationship model in interpreting the socioeconomic disparities in child health. The COVID-19 pandemic has pushed many families into poverty. Moreover, child mental health issues as an increasing public health problem have also been exacerbated [7]. Therefore, tackling the income gradient in child mental health is imperative. Given the robust relationship between income and child depression, and the fact that no one strategy has been successful in breaking the poverty cycle, this study demonstrates that an integration of interventions to improve distal social environments with income redistribution is necessary.

Concisely, to improve child mental health and reduce disparities, in addition to antipoverty strategies, social workers may consider an enhanced intervention on neighborhood cohesion and school social capital. In terms of neighborhood cohesion, children should be encouraged to participate in a variety of neighborhood activities to get to know other members, cultivate a sense of belonging and form neighborly relationships. Moreover, social workers should give extra care and support to left-behind children to ensure that this vulnerable group benefits equally from neighborhood resources. In terms of school social capital, school social workers could work on positive interactions between teachers and students. Similarly, special attention and care should be given to children from impoverished households and children with migrating parents.

This study has some limitations. First, confined by the survey, we used the aggregated data of adult respondents’ perceptions as the measurements of neighborhood social capital. In this case, the “modifiable area unit” problem may be encountered. Additionally, children, as active social agents, shape the structures around them and have their own experiences [60]. Therefore, future studies exploring children’s perceptions of social environments are needed. Second, this is a cross-sectional study. Therefore, a causal relationship cannot be established. Third, limited by the sample scale, we briefly categorized our respondents into left-behind and not-left-behind children. However, some children may have mixed experiences of being left behind and/or being migrants. Moreover, there are persistent urban–rural dual structures in China. On one hand, the nurturing of social capital components is different with urban ones being based mainly on contracts and regulations and rural ones being based mainly on kinship networks. On the other hand, although they share the same experience of prolonged parental absence and are both subject to a higher likelihood of psychological issues, urban and rural LBC might undergo different psychological processes amid varying social environments. Thus, differentiating children in urban areas from children in rural areas in examining the socioenvironmental mechanisms of the family income gradient among LBC and NLBC should bring more concise findings and insights. Future research should therefore, if possible, conduct analyses with a more concise categorization.

## Conclusions

There was a significant and robust family income gradient in child depressive symptoms. Being negatively associated with income and protective against depression, neighborhood cohesion mitigated the income gradient in child depression by playing a significant suppression effect. School social capital was a mediator through which income indirectly affected child depression. Neighborhood trust and safety and family social capital failed to play any mediating effect. The suppression role of neighborhood cohesion and the mediating role of school social capital were significant among only the not-left-behind children. This study enriches our understanding of the socioeconomic disparities in child health by empirically exploring the family income gradient in self-reported child depressive symptoms using Chinese nationally representative data and differentiating between left-behind and not-left-behind children. Moreover, by simultaneously considering material resources and neighborhood, home and school social environments in interpreting the income gradient in child depression, our study provides empirical support for the norms and collective efficacy model and a valuable reference for improving child mental health and reducing disparity.

## Supplementary Information

**Additional file 1:** **Table A.1.** Center for Epidemiological Studies Depression Scale for Children (CES-DC). **Table A.2.** Indicators of neighborhood social capital components.

## Data Availability

The data that support the findings of this study are available from the Institute of Social Science Survey of Peking University but restrictions apply to the availability of these data, which were used under license for the current study, and so are not publicly available. Data are however available from the corresponding author upon reasonable request and with permission of the Institute of Social Science Survey of Peking University.

## Abbreviations

LBC: Left-behind children
NLBC: Not left-behind children
CFPS: China Family Panel Studies
CES-DC: The Center for Epidemiological Studies Depression Scale for Children
SEM: Structural equation modeling
CR: Critical ratio
MCAR: Missing completely at random
RMSEA: The Root Mean Square Error of Approximation
CFI: The Comparative Fit Index
TLI: Tucker Lewis index
SRMR: Standardized Root Mean Square Residual

## Footnotes

1 χ^2^ is sensible to sample scales. When sample size is large, even a well-fitted hypothesized model may end up with a significant χ^2^ value.

2 Although plenty of resources have redeemed a Cronbach’s α value of above 0.7 to be acceptable, there are also studies that report 0.6 or above 0.64 to be adequate. It is worth noting that α is a function of the average interrelations and the number of items, and a very high α may indicate redundancy. Given we have small numbers of items, the α values here are acceptable.
