# Supplementary material for: Does social capital aid in leveling the income gradient in child mental health? A structural analysis of the left-behind and not-left-behind Chinese children
Source: BMC Public Health. 2023 Jul 20;23:1404. doi: 10.1186/s12889-023-16264-9 (PMC10360305; doi:10.1186/s12889-023-16264-9)
Supplement: Supplementary file 1 — Additional file 1: Table A.1. Center for Epidemiological Studies Depression Scale for Children (CES-DC). Table A.2. Indicators of neighborhood social capital components. [file 12889_2023_16264_MOESM1_ESM.docx]

**Table A.1.** Center for Epidemiological Studies Depression Scale for Children (CES-DC)

| **Below is a list of the ways you might have felt or acted. Please check how much you have felt this way during the past week.** | **Responses and scores** |
| --- | --- |
| 1. I was bothered by things that usually don’t bother me. | Not at all (0), A little (1), Some (2), A lot (3) |
| 2. I did not feel like eating, I wasn’t very hungry. | Not at all (0), A little (1), Some (2), A lot (3) |
| 3. I wasn’t able to feel happy, even when my family or friends tried to help me feel better. | Not at all (0), A little (1), Some (2), A lot (3) |
| 4. I felt like I was just as good as other kids. | Not at all (3), A little (2), Some (1), A lot (0) |
| 5. I felt like I couldn’t pay attention to what I was doing. | Not at all (0), A little (1), Some (2), A lot (3) |
| 6. I felt down and unhappy. | Not at all (0), A little (1), Some (2), A lot (3) |
| 7. I felt like I was too tired to do things. | Not at all (0), A little (1), Some (2), A lot (3) |
| 8. I felt like something good was going to happen. | Not at all (3), A little (2), Some (1), A lot (0) |
| 1. I felt that I have been a loser for a long time. | Not at all (0), A little (1), Some (2), A lot (3) |
| 10. I felt scared. | Not at all (0), A little (1), Some (2), A lot (3) |
| 11. I didn’t sleep as well as I usually sleep. | Not at all (0), A little (1), Some (2), A lot (3) |
| 12. I was happy. | Not at all (3), A little (2), Some (1), A lot (0) |
| 13. I was more quiet than usual. | Not at all (0), A little (1), Some (2), A lot (3) |
| 14. I felt lonely like I didn’t have any friends. | Not at all (0), A little (1), Some (2), A lot (3) |
| 15. I felt like kids I know were not friendly or that they didn’t want to be with me. | Not at all (0), A little (1), Some (2), A lot (3) |
| 16. I had a good time. | Not at all (3), A little (2), Some (1), A lot (0) |
| 17. I felt like crying. | Not at all (0), A little (1), Some (2), A lot (3) |
| 18. I felt sad. | Not at all (0), A little (1), Some (2), A lot (3) |
| 19. I felt people didn’t like me. | Not at all (0), A little (1), Some (2), A lot (3) |
| 20. It was hard to get started doing things. | Not at all (0), A little (1), Some (2), A lot (3) |

**Table A.2.** Indicators of neighborhood social capital components

| **Components** | **Indicators** | **Responses and scores** |
| --- | --- | --- |
| Neighborhood cohesion | How is the relationship between neighbors in your community? | 1 (very poor) to 5 (very good) |
|  | When you need any help from neighbors, will someone lend you a hand? | 1 (definitely NO) to 5 (definitely YES) |
|  | Are you emotionally attached to your neighborhood? | 1 (Not at all) to 5 (Very much) |
| Perception of trust | How much do you trust your parents? | 0 (Distrustful) to 10 (Very trustworthy) |
|  | How much do you trust your neighbors? |  |
|  | How much do you trust people you meet for the first time? |  |
|  | How much do you trust your doctors? |  |
|  | How much do you trust the local government officials? |  |
| Perception of safety | How do you rate public safety in your neighborhood? | 1 (very poor) to 5 (very good) |
